# An Investigation on the Comprehensive Property Assessment and Future Directions of Single Bamboo Fiber Reinforced Polypropylene Composites Fabricated by a Non-Woven Paving and Advanced Molding Process

**DOI:** 10.3390/ma12162641

**Published:** 2019-08-19

**Authors:** Qiheng Tang, Yunfei Wang, Ge Wang, Haitao Cheng, Wenjing Guo

**Affiliations:** 1 Research Institute of Forestry New Technology, Chinese Academy of Forestry, Xiangshan Road, Beijing l00091, China; 2 Research Institute of Wood Industry, Chinese Academy of Forestry, No. 1 Dongxiaofu, Haidian District, Beijing 100091, China; 3 Department of Biomaterials, International Center for Bamboo and Rattan, No. 8, Futong Eastern Street, Wangjing Area, Chaoyang District, Beijing 100102, China

**Keywords:** composites, single bamboo fiber, mechanical property, thermal property

## Abstract

The demand for eco-friendly renewable natural fibers has grown in recent years. In this study, a series of polypropylene-based composites reinforced with single bamboo fibers (SBFs), prepared by non-woven paving and a hot-pressing process, were investigated. The influence of the content of SBF on impact strength, flexural strength, and water resistance was analyzed. The properties of the composites were greatly affected by the SBF content. Impact strength increased as SBF content increased. The modulus of rupture and modulus of elasticity show an optimum value, with SBF contents of 40% and 50%, respectively. The surface morphology of the fractured surfaces of the composites was characterized by scanning electron microscopy. The composites showed poor interfacial compatibility. The water resistance indicates that the composites with higher SBF contents have higher values of water absorption and thickness swelling, due to the hydrophilicity of the bamboo fibers. The thermal properties of the composites were characterized by thermal gravimetric analysis and by differential scanning calorimetry. The thermal stability of the composites was gradually reduced, due to the poor thermal stability of SBFs. In the composites, the maximum decomposition temperature corresponding to SBF shows an increasing trend. However, the maximum decomposition temperature of polypropylene was not influenced by SBF content. The melting point of the polypropylene in the composites was lower relative to pure polypropylene, although it was not affected by increasing SBF content.

## 1. Introduction

Man-made fiber-reinforced polypropylene (PP) composites are widely used in industry because of their high specific strength and stiffness. The manufacture of fibers of carbon [1,2], glass [3,4], and aramid [5] is energy consuming and causes environmental pollution and the depletion of resources. With the increasing awareness of environmental protection, environmental policies that promote the use of composites derived from recycling plants have provided the incentive to find new products that are compatible with the environment.

An alternative approach to man-made fibers is to use naturally renewable and biodegradable plant fibers as a reinforcement for polymer-based composites. This alternative has drawn much interest in recent years. Plant fibers possess the advantage of a high aspect ratio and a high strength to weight ratio [6,7,8,9]. These advantages make them a potential replacement for man-made fibers in fiber-reinforced composites. Compared with man-made fibers, the majority of plant fibers have relatively low mechanical properties [10,11], which are shown in Table 1.

Plant fibers fall into various categories, including ramie, flax, sisal, wood, and grass fibers. Because of their environmentally friendly characteristics, composites reinforced with plant fibers have been widely used in automotive interiors, furniture, and landscape gardening [12,13]. Bamboo (Bambusoideae, the grass family Poaceae) is particularly abundant in Asia, especially in China. Bamboo fibers have many advantages over other plant fibers, including high mechanical strength, high growth rate, and the ability to fix atmospheric carbon dioxide [14,15,16]. Moreover, because bamboo fibers are composed of elementary fibers that are aligned longitudinally, they are called “natural glass fibers” [17]. This advantage makes bamboo fibers an ideal replacement for man-made fibers for the production of fiber-reinforced composites. Due to its high strength to weight ratio, bamboo has been traditionally used in construction and as a material for manufacturing tools.

At present, composites reinforced with bamboo are being studied in Asia but not in America or Europe, due to the lack of bamboo resources. Many studies have focused on various topics, such as the use of bamboo fibers [12,18], bamboo particles [19], bamboo flours [20], and bamboo chopsticks [21], to reinforce polypropylene, poly lactic acid, or other polymers. 

Nahar et al. [22] studied PP-composites reinforced with jute and bamboo fibers (50 wt.% fiber) fabricated by compression molding. The resulting bamboo fiber-based composites had a higher tensile strength, higher bending strength, higher tensile modulus, and higher bending modulus compared to jute-based composites. Sukmawan et al. [23] investigated the mechanical properties of poly lactic acid reinforced with bamboo fibers. The tensile strength of the composite was comparable to that of ordinary glass fiber-reinforced plastics. Moreover, the specific strength of this composite was three times as high as that of mild steel. 

Li et al. [24] studied the effect of treating the surface of bamboo fibers on the properties of bamboo-fiber/nanohydroxyapatite/poly(lactic-co-glycolic) composites. Bamboo fibers (BFs) surface-treated with alkali increased their tensile and bending properties. Liu et al. [25] studied the effect of bamboo fibers reinforcing grafted acrylated epoxidized soybean oil (AESO) on unsaturated polyester. The grafting of BFs with AESO resulted in improved tensile and flexural properties, storage modulus, and thermal stability of the composite.

**Figure 1:**
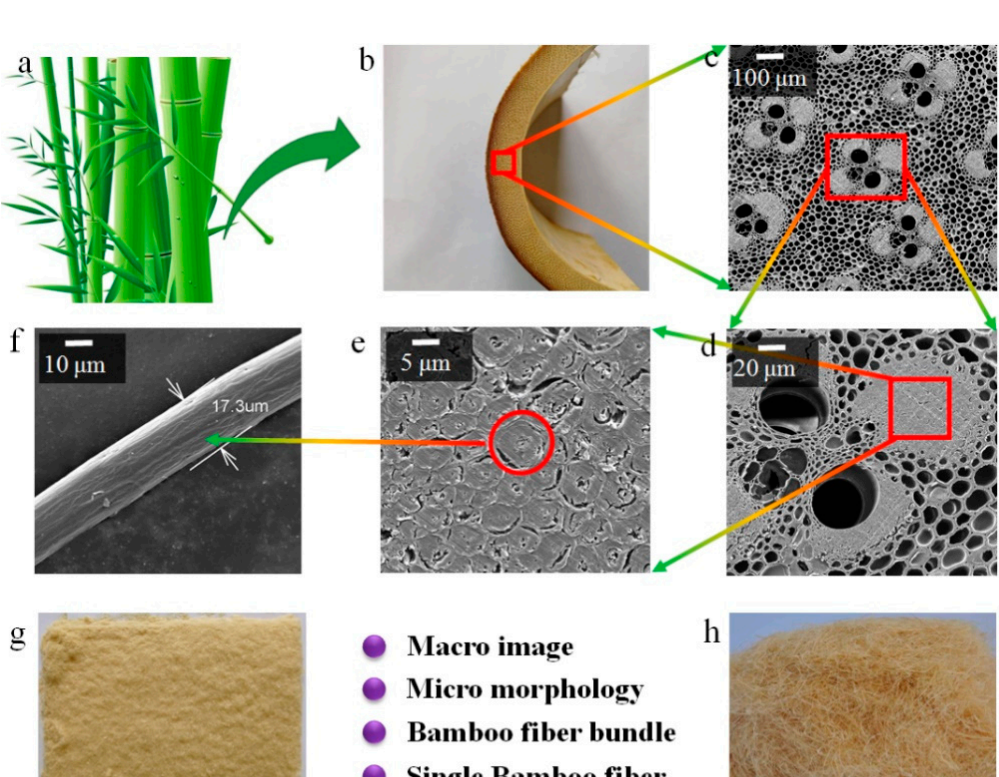
Macro images and micro morphology of bamboo: (**a**) bamboo, (**b**) cross section of cylindrical bamboo culm, (**c**–**f**) distribution of bamboo fiber in bamboo culm, (**g,h**) macro images of the single bamboo fibers (SBFs) and bamboo fiber bundle.

The bamboo fibers previously described in the literature are used at the fiber bundle level (Figure 1e,g). This means that there are many interfaces and gaps in the fiber bundles. In addition, multiple defects of fiber bundles inhibit the transfer of stress, resulting in poor mechanical properties of the composites.

Bamboo fiber bundles consist of multiple single bamboo fibers (SBFs) (Figure 1d–f). Yu et al. [26] and Chen et al. [27] have reported that single bamboo fibers have excellent mechanical properties. The tensile strength and modulus of single fibers of Moso bamboo are approximately 1.55 GPa and 36.7 GPa, respectively. The tensile strength value is not only higher than that of bamboo fiber bundles (about 500 MPa [28]), but can also be close to the tensile strength of the glass fiber [16].

Most of the deterioration of the mechanical properties of laminated composite materials result from delamination. Poor interlaminar shear results from the lack of a Z-direction in the inter ply fiber reinforcement between the assembled composite laminar plies [29]. Non-woven airflow paving technology is a common method to evenly mix various fibers in the X, Y, Z directions. X includes a fiber loosening machine, an air-flow netting machine, and a needle machine. First, the fibers are loosened, and the loose fibers are then mixed and conveyed to a high vibration pneumatic chamber by air. Second, the mixed fibers are evenly conveyed to the air-flow netting machine through the vibration feeding function of the vibration chamber, and then a three-dimensional fiber network with uniform mixing and non-directional arrangement is achieved. Lastly, the three-dimensional fiber networks are needled by a needle machine to obtain fiber mats.

Previous research only dealt with composites reinforced with fiber bundles but did not consider using single fibers as reinforcement for composites. In this study, we propose to use single bamboo fibers to reinforce PP composites to improve the mechanical properties of composites expected to be used in semi-structural areas in automobiles. Therefore, a non-woven air paving technology was used to prepare SBF/PP mats, which not only contributed to the content of fibers in the Z-direction, but also improved the uniform distribution of the fibers. SBF/PP composites were prepared by hot-pressing the mats. 

To evaluate the influence of single fiber content on the properties of SBF-reinforced PP composites, their mechanical properties and thermal behavior were characterized by impact strength, bending strength, thermal gravimetric analysis (TGA), and differential scanning calorimetry (DSC). The microstructural morphology and water absorption of the composites were also investigated and compared to composites reinforced with fiber bundles. 

## 2. Materials and Methods 

### 2.1. Materials

SBFs were obtained from Fujian Haibosi Chemical Technology Co. Ltd. (Fujian, China). The diameter of the fibers was 10–20 μm, and their length was about 0.5 cm. PP fibers were bought from Taizhou Hailun Chemical Fiber Co. Ltd. (Guangdong, China). Their linear density was 11.11 dtex, and their length was 6–8 cm. Hydrogen peroxide (H_2_O_2_), sodium hydroxide (NaOH), sodium metasilicate (Na_2_SiO_3_), and 1,4-butanediol were from Beijing Chemicals Co. Ltd. (Beijing, China). 

### 2.2. Fiber Preparation

Bamboo (2–3 years old) was cut into strips 10–20 mm wide. These strips were soaked, milled, and softened. The original bamboo fibers bundles were then separated into single fibers using a steel comb. The detail production process is as follows. The cylindrical bamboo culms, excluding the node portions, were cut into smaller 10 to 20 mm wide chips. The chips were placed into a digester that was charged with 70% 1,4-butanediol water solution, and digestion was carried out at 170 °C for 10 h to remove the lignins. Chips were rolled into bamboo threads through a roller. The bamboo threads were delignified and bleached by using a mixture of H_2_O_2_ (6%), NaOH (3%), and Na_2_SiO_3_ (2%) solutions at 100 °C for 2 h. The threads were washed several times in distilled water and dried at room temperature. Finally, SBFs were obtained by carding. The Macro photographs and micro morphology of the SBFs are shown in Figure 1.

### 2.3. Composite Preparation

**Figure 2:**
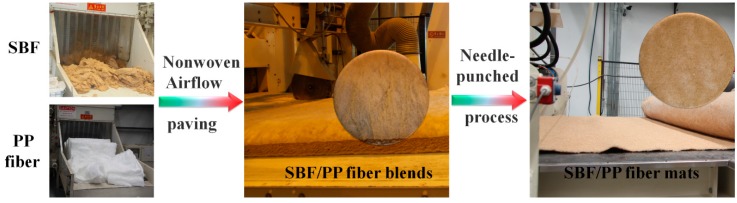
Graphical illustration of the mat production from SBFs and polypropylene (PP) fibers.

**Figure 3:**
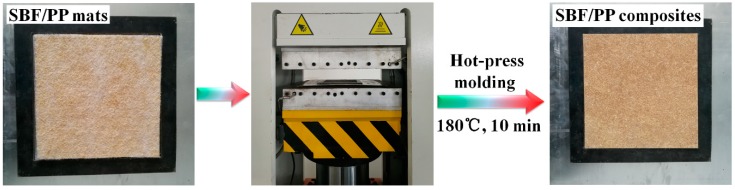
Graphical illustration of SBF/PP composites.

SBFs and PP fibers were mixed by non-woven air paving technology to produce fiber SBF/PP mats (Figure 2). The mat was then placed in a 22 cm × 22 cm × 0.4 cm mold. The mold was hot-pressed at 10 MPa and 180 °C for 10 min, allowing the PP fibers to melt. After hot-pressing, the mold was pressed at room temperature, and the SBF-reinforced composite panels were removed from the mold. The details of the hot-press molding are shown in Figure 3. The weight fractions of SBFs in the total weight composites were 10 wt.%, 20 wt.%, 30 wt.%, 40 wt.%, 50 wt.%, and 60 wt.%.

### 2.4. Characterization and Measurement 

#### 2.4.1. Tests of Mechanical Properties

Tests of mechanical properties were performed using a universal testing machine (Zwick/Roell Z030, Zwick/Roell, Ulm, Germany). 

The bending properties of the composites were tested by a three-point bending method following the previously published ISO 16978: 2003 [30]. The flexural test was carried out at room temperature with a load rate of 10 mm/min. The dimension of the specimens was 150 × 50 × 4.0 mm^3^. Five specimens were tested.

Impact tests were performed on samples with dimensions of 80 × 10 × 4.0 mm^3^, using a universal testing machine (XJJ-5, Jinan Zhongte Testing Machine Co., Ltd., Shandong, China), according to GB/T1043.1-2008 [31]. Five specimens were tested.

#### 2.4.2. Scanning Electron Microscopy (SEM)

The morphology of the composites was observed by SEM. Samples were introduced in liquid nitrogen and fractured. The fractured surfaces were subjected to gold sputtering and observed using a JEOL JM-6400 microscope (JEOL Ltd., Tokyo, Japan) operating at 40 kV.

#### 2.4.3. Thermal Gravimetry (TG)

TG was performed using a Mettler Toledo TGA/DSC1 TG apparatus (Mettler Toledo, Zurich, Switzerland). Samples were placed in an aluminum crucible of 70 mL and heated at 10 °C/min over a temperature range of 30–600 °C. The experiments were performed in a nitrogen atmosphere at a flow of 20 mL/min.

#### 2.4.4. Differential Scanning Calorimetry (DSC)

The DSC of PP was characterized using a Mettler Toledo TGA/DSC1 DSC apparatus (Mettler Toledo, Zurich, Switzerland). Samples (5–10 mg) were loaded and sealed in an aluminum pan. The thermograms of samples were obtained by heating from 25 to 300 °C at 10 °C/min in a nitrogen atmosphere. 

#### 2.4.5. Water Absorption (WA) and Thickness Swelling (TS) Properties

WA and TS were evaluated from the difference between the specimen’s thickness and weight, both before and after immersion in water for 24 h, according to ISO 16983:2003 [32]. The sample was 50 × 50 × 4.0 mm^3^. Five specimens were tested, and the values of WA and TS are the average of the five samples.

The ratios of WA and TS were calculated using Equations (1) and (2), respectively:

(1) $$\mathrm{WA}(\%)=\frac{m_{2}-m_{1}}{m_{1}}\times 100\%,$$

(2) $$\mathrm{TS}(\%)=\frac{h_{2}-h_{1}}{h_{1}}\times 100\%,$$

where *m_2_* and *h_2_* are the final weight and thickness after immersion in water, respectively; *m_1_* and *h_1_* are the initial weight and thickness before immersion in water, respectively. 

## 3. Results and Discussion

### 3.1. Impact Strength Analysis

**Figure 4:**
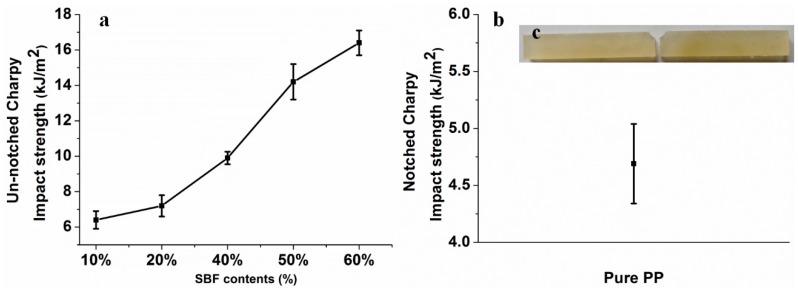
Impact strength of the composites and pure PP: (**a**) the un-notched charpy and (**b**) notched charpy of PP; (**c**) image of the PP after the impact test.

Plant fiber composites are widely used in automotive interiors, where the shock resistance of composites plays an important role. Data on the impact strength of the composites are summarized in Figure 4a,b, which provides clear insight into the shock resistance properties of pure PP and SBF/PP composites. Impact strength shows a significant variation compared to other composites. Furthermore, the strength values increase monotonically as SBF content increases, meaning that the composite toughness increases as fiber content increases. The impact strength of the SBF/PP composites with 10 wt.% fiber was 6.4 kJ/m^2^. When the fiber content was 60 wt.%, the impact strength was 16.4 kJ/m^2^. Thus, in the composites, the impact strength increased by 156.2%, relative to composites with 10 wt.% SBFs. Pure PP exhibits very good toughness. The impact strength cannot be characterized by an un-notched charpy impact test. The notched charpy impact strength of PP is 4.69 kJ/m^2^, and the image of PP after the impact test is shown in Figure 4c. 

**Figure 5:**
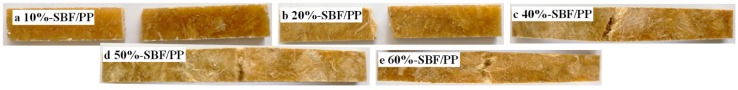
Photograph of the composites after the impact test. (**a**–**e**) belong to the composites with 10%–60% SBF, respectively.

The images of the composites after impact tests are shown in Figure 5. The composites with 10 wt.% and 20 wt.% SBFs split after the impact test. After that, composites with other fiber contents broke but did not split, indicating that composites with higher fiber contents exhibit higher toughness. This phenomenon can be attributed to the high mechanical properties of the SBFs. More SBFs are disrupted, which contributes to the high impact strength.

In comparison with the composites reinforced with bamboo fiber bundles, the SBF/PP composites show excellent impact strength. Relative to the maximum values of the impact strength of the composites reinforced with bamboo fiber bundles (9.1 kJ/m^2^ [33] or 3.8 kJ/m^2^ [34]), the maximum value for the SBF/PP composites (16.4 kJ/m^2^) increased 80.22%–331.58%.

The strength of the SBF/PP composites with 60 wt.% fiber is 43.36% higher than that in our previous report (11.44 kJ/m^2^) [12]. This improvement is attributed to the better mechanical properties of SBFs compared to the bamboo fiber bundles.

### 3.2. Bending Properties Analysis

**Figure 6:**
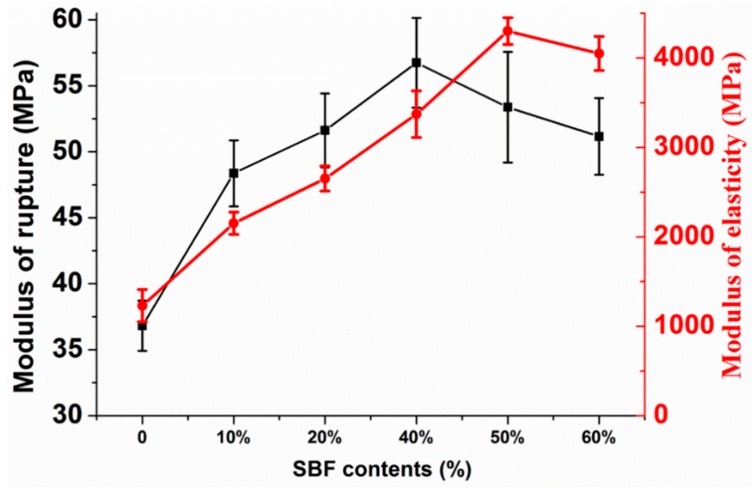
The modulus of rupture (MOR) and modulus of elasticity (MOE) of the pure PP and composites with different SBF contents.

The bending properties of pure PP and composites are characterized by the modulus of rupture (MOR) and the modulus of elasticity (MOE). The variation of the MOR and MOE for the composites with different fiber contents is shown in Figure 6. The MOR and MOE of pure PP are 36.84 MPa and 1160 MPa, respectively. As expected, the MOR and MOE of the composites improved due to the enhancement effect of the SBFs. Moreover, the composites exhibit optimum values (56.75 MPa and 4300 MPa) when the SBF contents are 40 wt.% and 50 wt.%, respectively. The optimum values of MOR and MOE increase 17% and 100%, relative to those of the SBF-10%/PP composites, respectively. This increment can be attributed to the inclusion of rigid SBFs into the composites. These SBFs have a reinforcing effect on the soft PP matrix, which is consistent with the results reported by Chen et al. [35]. Subjected to MOE, the matrix mobility is reduced by the incorporation of fiber, so the stiffness of composites increases as the fiber content increases. SBFs are a high modulus material. A higher loading content means higher stress for the composites with the same level of deformation. 

However, increasing the content of SBFs leads to a gradual decrease of the MOR and MOE. This may be because the fibers act as defects in the composites when the content of SBFs exceeds the limit. At high fiber concentrations, fibers are not sufficiently wetted by the matrix (a smaller amount), and this results in lower interface adhesion between the SBFs and PP, such that ineffective stress is transferred from the matrix to the fiber [36]. This can be confirmed by the fact that a high concentration of agro waste fiber requires higher amounts of a coupling agent to provide better adhesion and mechanical properties [37].

**Figure 7:**
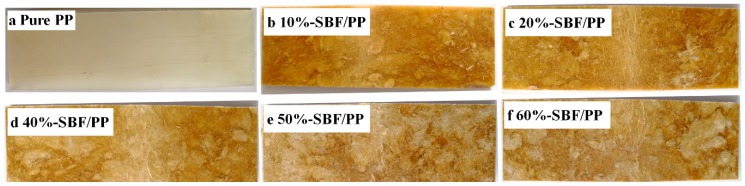
Photographs of the pure PP and composites after bending tests. (**a**–**e**) belong to the pure PP and composites with 10%–60% SBF, respectively.

Optical images of pure PP and SBF/PP composites, taken after the bending test, show that PP and the composites were bent but did not split (Figure 7). According to the above analysis and the photos, pure PP and the SBF/PP composites exhibit excellent bending resistance. The optimum MOR value is higher than the highest MOR of the composites reinforced with bamboo fiber bundles, as reported by Chattopadhyay et al. [38] and Rahman et al. [39] (49.6 MPa and 39.0 MPa, respectively). The maximum MOE that has been reported is 3.3 GPa for a 60% volume content of bamboo fiber [38] and 3.2 GPa for a 25% weight content of BF [39]. Compared to the optimum value in this study, the values reported by Chattopadhyay et al. [38] and Rahman et al. [39] are lower by 30% and 34%, respectively. These results indicate that SBF/PP composites have potential applications in industry.

### 3.3. SEM Analysis

**Figure 8:**
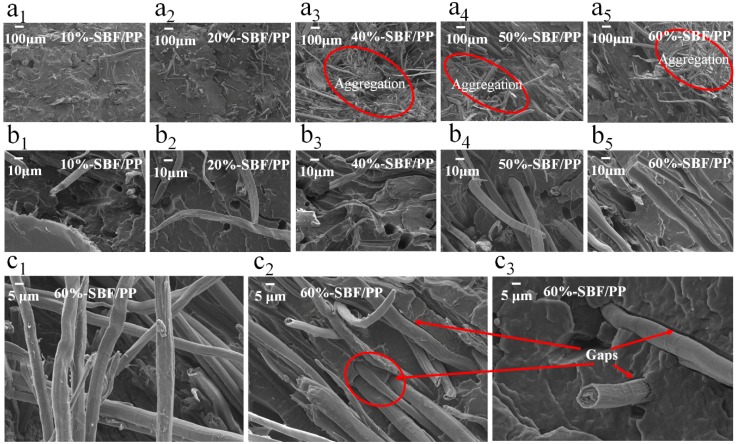
SEM images of impact-fractured surfaces of composites. (**a_1_**–**a_5_**) SBF/PP with 10% to 60% SBF, magnification 50×. (**b_1_**–**b_5_**) SBF/PP with 10% to 60% SBF, magnification 500×. (**c_1_**–**c_3_**) SBF/PP with 60% SBFs, magnification 1000×.

The morphology of the impact fracture surfaces of the SBF/PP composites is shown in Figure 8. Representative SEM images of the fracture surfaces are shown in Figure 8a–c. At low magnification (Figure 8a_1_,a_2_), it was clear that the SBFs were distributed uniformly in the matrix. However, as the fiber content increases (Figure 8a_3_,a_4_), the fibers gradually aggregate. Aggregation may be due to the large special areas of SBFs, which result in large interaction forces between the two SBFs. Figure 8b_1_–b_5_ shows the fracture at higher magnification. It can be observed that numerous SBFs are pulled out from the PP resin, leaving holes at the fracture surface. This indicates that in the SBF/PP composite, there is poor interfacial adhesion between the fiber and the matrix. This is due to the poor compatibility between the non-polar PP and polar SBFs with numerous hydroxyl groups.

To better explain the problem, the SBF-60%/PP composite was taken as an example. The micro morphology of the composites at 1000× magnification is shown in Figure 8c_1_–c_3_. There are numerous gaps between the SBFs and PP, and the surfaces of the pulled fiber are smooth. It can be inferred that if the SBFs were to be modified to improve the interface compatibility, their mechanical properties could be hugely improved.

### 3.4. Analysis of Thermal Properties

**Figure 9:**
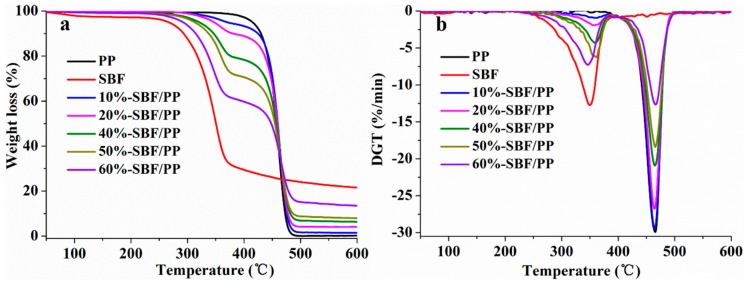
Thermogravimetric analysis (**a**) and derivative thermogravimetric analysis (**b**) curves of PP, SBFs, and the composites with different SBF content.

**Figure 10:**
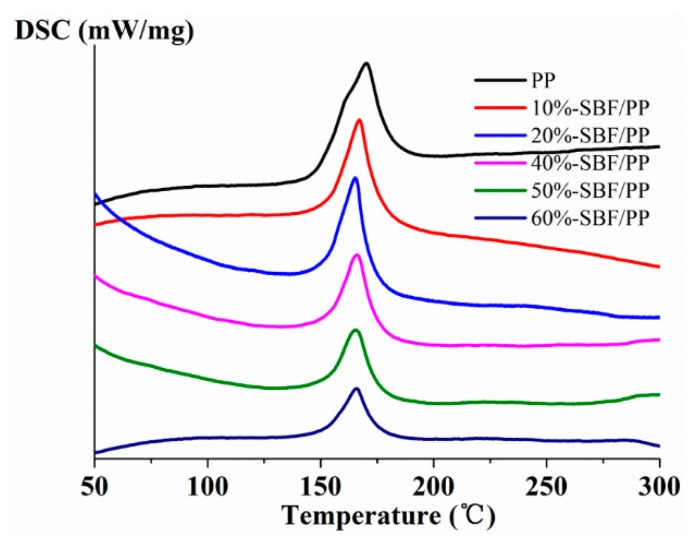
Differential scanning calorimetry (DSC) curves of PP and the composites with different SBF contents.

The thermal properties of the composites reinforced with natural fibers have a large effect on their processing and practical applications. Limited thermal stability during the melting processing constitutes a barrier in the manufacture of composites. The thermal stability and crystallization behavior of composites are often characterized by TG and DSC. The relevant curves of the composites with different SBF contents are shown in Figure 9 and Figure 10. Detailed data are presented in Table 2. T_5%_ is defined as the initial decomposition temperature of the composites with a weight loss of 5%. T_maxs1_ and T_maxs2_ are defined as the decomposition temperatures of the maximum weight loss. T_c_ is defined as the temperature of the melting-point of PP.

#### 3.4.1. TGA

The TGA and derivative thermogravimetric analysis (DTG) curves of PP, SBFs, and composites are shown in Figure 9a,b, respectively. The thermal decomposition of the PP shows only one step at 463.0 °C and remains 0.04% chars at 600 °C, and T_5%_ is 415.2 °C. For SBFs, the TG curve consists of two steps of thermal degradation. The initial decomposition peak T_5%_ appears at 266.9 °C, and the maximum decomposition peaks are at 349.7 °C. The first step takes place at approximately 100 °C and corresponds to moisture loss and loss of volatiles (e.g., formaldehyde). The second step (250–350 °C) corresponds to the degradation of cellulose. The cellulose fibers are degraded in one step (250–400 °C), in which the cellulose decomposes through the formation and decomposition of levoglucosan [40,41]. The decomposition of polymer chains occurs, and water and formaldehyde are formed and released.

The decomposition of the composites occurs in two steps: the decomposition of SBFs and the decomposition of PP. According to Table 2, due to the poor thermal stability of SBFs, the initial decomposition temperature of the composites decreases as SBF content increases, which means that the thermal stability of the composites decreases gradually. For the composites with 10–50 wt.% SBFs, the T_max1_ increases with SBF content. This may be due to the PP matrix with higher thermal stability absorbing the heat and inhibiting the degradation of SBFs. However, for SBF-60%/PP, the Tmax1 was low because the composite contains excessive SBFs, and the PP content could not inhibit the degradation of SBFs. When the SBF contents changed, there were no changes in T_max2_, indicating that SBFs have no effect on the decomposition of PP. The char at 600 °C increases with SBF content, which is tied to the high char of SBFs.

#### 3.4.2. DSC Analysis

DSC is a common method to investigate the melting behavior of polymers. The PP fiber used in the study is isotactic polypropylene, which is a crystalline polymer. Therefore, a DSC calorimeter was used to analyze the crystallization behavior of PP and SBF/PP composites in a nitrogen atmosphere. To investigate the practical situation of the composites, every sample was scanned at a constant heating rate of 10 °C /min from 50 °C to 300 °C, without removing the previous thermal history. The details of the thermal data are shown in Figure 10 and Table 2. The PP melted at 170.3 °C. After adding SBFs, the melting-point of the composites gradually decreased. This decrease may be due to the physical effects, which are the molecular chains of the PP not being orderly arranged under the restriction of SBF [33]. SBFs act as a restriction site for PP, which could not promote the growth of the crystal. For the composites with 20–60 wt.% SBFs, Tc did not change with SBF content. It can be inferred that SBFs did not significantly affect the melting temperature of pure PP at a high fiber content. The melting enthalpy of SBF/PP decreased as the SBF content increased because the PP content in the composites gradually decreased.

### 3.5. Water Resistance Analysis

**Figure 11:**
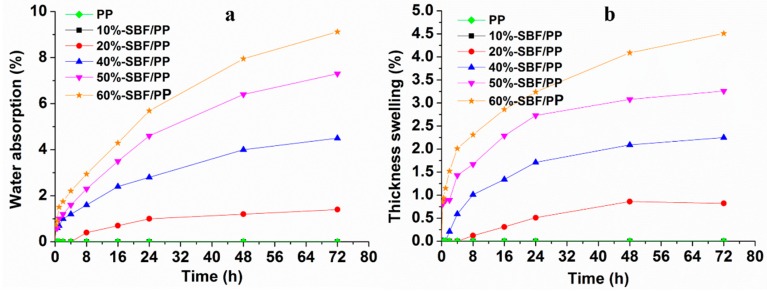
Water resistance of the pure PP and composites with different SBF contents. (**a**) waster absorption, (**b**) thickness swelling.

Water resistance behavior is an important physical property of the composites reinforced with plant fibers. Therefore, the effects of fiber content on WA and TS were assessed (Figure 11). The WA (Figure 11a) and TS (Figure 11b) of the composites increase with fiber content, which can be attributed to the interaction between the water molecules and SBFs. When the content of SBFs increases in the composites, there are more hydroxyl groups available to combine with the water molecules to form hydrogen bonds. The immiscibility of SBFs and PP resin leads to the formation of voids or microgaps (Figure 8) between the interfaces of both components and improves the penetration and diffusion of water molecules [36]. Another possible reason for this result is the hydrophilicity of SBFs, which is responsible for the water absorption in the biocomposites [42].

Figure 11 shows that the composites with 20 wt.%–60 wt.% SBFs have a rapid increase in TS (Figure 11b) before 24 h. After this time, TS increases slowly. These composites have low TS (below 3%) from 0–72 h. Because PP is the main component of the composites, the bamboo fibers can be coated, which contributes to inhibiting the permeation of water into the composites. This occurs because the composite has an excess of SBFs. Thus, the SBF-60%/PP needs more time to reach the saturation point.

## 4. Conclusions

SBFs are an effective reinforcement to improve the mechanical properties of composites. The impact strength and MOE increased with SBF content, giving composites toughness and rigidity. Moreover, the optimum values of MOR and MOE increased by 17% and 100%, respectively, relative to the 10%-SBF/PP composites. This enhancement of performance will expand the applications of SBF reinforced composites in industries such as the automotive industry and housing/construction. SEM analysis indicates that the composites have poor interfacial compatibility after the surfaces are modified, which leaves room for improvement of the mechanical properties. The addition of SBFs to PP is a promising way to improve its mechanical properties. The thermal stability and eutectic point of the SBF composites was reduced compared with the pure PP. However, the maximum decomposition temperature and melting point of the composites did not change with SBF content. The WA and TS of the composites increase gradually as the SBF content and immersion time increases, due to the hydrophilic character of SBFs.

## Figures and Tables

**Table 1:** The physical properties of natural fibers compared with conventional synthetic fibers.

Fiber	Tensile Strength (MPa)	Elongation at Break (%)	Young’s Modulus (MPa)	Density (g/cm^3^)
Jute	187–773	1.4–3.1	3–55	1.30–1.50
Flax	343–1035	2.7–3.2	27–80	1.40–1.50
Sisal	507–855	2.0–2.9	9.0–28.0	1.30–1.50
Ramie	400–938	3.6–3.8	44–128	~1.50
Hemp	580–1110	1.3–4.7	3–90	1.40–1.50
E-glass	2000–35,000	~2.5	~73.0	2.50–2.55
Carbon	~4000	5.5–6.9	230.0–240.0	1.40–1.75

**Table 2:** Thermal properties of PP, SBFs, and the composites.

Samples Name	T_5%_ (°C)	T_maxs1_ (°C)	T_maxs2_ (°C)	Char at 600 °C (%)	T_c_ (°C)	Melting Enthalpy (J/g)
PP	415.2	463.0	-	0.04	170.3	102.5
SBFs	266.9	-	349.7	21.7	-	-
10%-SBF/PP	371.0	349.6	464.6	1.5	167.1	79.1
20%-SBF/PP	347.3	356.3	464.2	3.6	165.2	76.9
40%-SBF/PP	320.8	359.5	465.0	6.4	165.9	68.3
50%-SBF/PP	312.3	360.9	465.7	8.2	165.4	58.8
60%-SBF/PP	292.3	346.2	466.1	13.4	165.8	49.3

T_5%_ is the temperature of the samples with a weight loss of 5%; T_maxs1_ and T_maxs2_ are the decomposition temperatures of the maximum weight loss; T_c_ is the melting point of PP.
